# Gla-Rich Protein, Magnesium and Phosphate Associate with Mitral and Aortic Valves Calcification in Diabetic Patients with Moderate CKD

**DOI:** 10.3390/diagnostics12020496

**Published:** 2022-02-15

**Authors:** Ana P. Silva, Carla S. B. Viegas, Patrícia Guilherme, Nelson Tavares, Carolina Dias, Fátima Rato, Nélio Santos, Marília Faísca, Edgar de Almeida, Pedro L. Neves, Dina C. Simes

**Affiliations:** 1 Department of Nephrology, Centro Hospitalar Universitário do Algarve, 8000-386 Faro, Portugal; anapassionara@gmail.com (A.P.S.); pleaon@hotmail.com (P.L.N.); 2 Department of Biomedical Sciences and Medicine, Universidade do Algarve, 8005-139 Faro, Portugal; heycarol.5@gmail.com; 3 Centre of Marine Sciences (CCMAR), Universidade do Algarve, 8005-139 Faro, Portugal; caviegas@ualg.pt; 4 GenoGla Diagnostics, Centre of Marine Sciences (CCMAR), Universidade do Algarve, 8005-139 Faro, Portugal; 5 Department of Cardiology, Centro Hospitalar Universitário do Algarve, 8000-386 Faro, Portugal; cpguilherme@gmail.com (P.G.); nelson.tavares63@gmail.com (N.T.); 6 Pathology Clinic, Centro Hospitalar Universitário do Algarve, 8000-386 Faro, Portugal; fatima.rato@gmail.com (F.R.); neliofilipe.santos@gmail.com (N.S.); marilia.faisca@synlab.pt (M.F.); 7 Centro Cardiovascular da Universidade de Lisboa (CCUL), 1649-028 Lisboa, Portugal; edealmeida@mail.telepac.pt

**Keywords:** chronic kidney disease, cardiovascular calcification, valvular calcification, vascular calcification, cardiovascular disease, Gla-rich protein, cardiovascular risk assessment

## Abstract

Accelerated and premature cardiovascular calcification is a hallmark of chronic kidney disease (CKD) patients. Valvular calcification (VC) is a critical indicator of cardiovascular disease and all-cause mortality in this population, lacking validated biomarkers for early diagnosis. Gla-rich protein (GRP) is a cardiovascular calcification inhibitor recently associated with vascular calcification, pulse pressure, mineral metabolism markers and kidney function. Here, we examined the association between GRP serum levels and mitral and aortic valves calcification in a cohort of 80 diabetic patients with CKD stages 2–4. Mitral and aortic valves calcification were detected in 36.2% and 34.4% of the patients and associated with lower GRP levels, even after adjustments for age and gender. In this pilot study, univariate, multivariate and Poisson regression analysis, show that low levels of GRP and magnesium (Mg), and high levels of phosphate (P) are associated with mitral and aortic valves calcification. Receiver operating characteristic (ROC) curves showed that the area under the curve (AUC) values of GRP for mitral (0.762) and aortic (0.802) valves calcification were higher than those of Mg and P. These results suggest that low levels of GRP and Mg, and high levels of P, are independent and cumulative risk factors for VC in this population; the GRP diagnostic value might be potentially useful in cardiovascular risk assessment.

## 1. Introduction

Chronic kidney disease (CKD), diabetes mellitus, and atherosclerosis are the clinical conditions that most contribute towards the development of cardiovascular calcification [1,2], which is a strong predictor of cardiovascular risk, while cardiovascular disease (CVD) is the most common cause of death in CKD patients [3]. Cardiovascular calcification can occur at different sites within the vascular tree, including the vessel wall at the media or intimal layers, and heart valves, with different impacts on cardiovascular outcomes. Intimal calcification reflects atherosclerotic plaque burden, may influence plaque rupture, and is a strong predictor of cardiovascular events and mortality [4]. Medial calcification induces stiffening of the vessel, increased pulse pressure and left ventricular hypertrophy, and can result in heart failure [5,6]. Valvular calcification causes valve stenosis, and can lead to cardiac hypertrophy, valve and heart failure, and sudden cardiac death [6,7]. In fact, accelerated and premature cardiovascular calcification is a hallmark of CKD patients, and all forms of calcification contribute to an increase in cardiovascular mortality in CKD [2,8]. Valvular calcification (VC) has been shown as a critical indicator of CVD and all-cause mortality in patients with CKD [9,10,11], occurring 10–20 years earlier in CKD patients when compared with the general population, and with incidence increasing with CKD progression [11,12,13,14,15]. Patients with CKD and VC were suggested to be considered at the highest CVD risk by the Kidney Disease: Improving Global Outcomes (KDIGO) CKD-Mineral and Bone Disorder (MBD) Work Group [16]. Although all cardiac valves can be affected, epidemiologic data on valvular calcification in CKD patients have been mainly reported for the mitral and aortic valves [8,13,17]. Mitral annular and aortic valve calcifications commonly originate valvular stenosis and regurgitation, conduction system abnormalities and endocarditis, which are associated with significantly reduced survival of CKD patients [13,17,18]. Additionally, symptomatic valve disease is preceded by a potentially long asymptomatic preclinical phase, challenging early detection and requiring constant monitoring in high risk patients [19]. Echocardiography remains the gold standard for diagnosis and evaluation of valvular disease, but with poor predictive value for the progression rate from the early to late stage of disease, and providing limited insights into disease pathophysiology. In this field, the discovery of early biomarkers related to specific pathophysiological mechanisms involved in disease pathogenesis would allow insights into causal factors, and improve time of intervention and risk stratification. It is known that VC share many biological pathways and pathophysiological mechanisms with vascular calcification, which is the most studied form of cardiovascular calcification [8,13]. Among the many factors involved in the pathogenesis or progression of VC, such as genetics, mechanical stress, metabolic factors and inflammation, and the use of drugs such as calcium supplements and calcium-based phosphate binders, a crucial role has been attributed to the osteochondrogenic differentiation of resident cells, and abnormalities of mineral and bone metabolism and hormonal-related factors [8,13]. Gla-rich protein (GRP), also known as upper zone of growth plate and cartilage matrix associated protein (UCMA) is a circulating vitamin K-dependent protein (VKDP) functioning as an inhibitor of vascular and valvular calcification with anti-inflammatory properties [20,21,22,23]. Recently, we have shown that in a cohort of 80 adult diabetic patients with mild to moderate CKD, serum GRP levels progressively decrease from stage 2 to stage 4 CKD, correlating with markers of mineral metabolism, and strongly associated with vascular calcification and pulse pressure [24]. The results strongly suggested GRP as a potential novel cardiovascular risk factor in this population. In the present study we explored, for the first time, the relationship between levels of circulating GRP and aortic and mitral valve calcification in the same cohort of diabetic patients with mild to moderate CKD (stage 2–4).

## 2. Materials and Methods

### 2.1. Patients 

This cross-sectional pilot study was conducted in the outpatient diabetic nephropathy clinic of the Centro Hospitalar Universitário do Algarve in Faro, Portugal, from 2012 to 2017, which is the reference centre for nephrology in the Algarve region. Out of the 107 recruited patients, 27 were excluded because they did not meet the inclusion criteria [24], leaving a total of 80 patients for the present study. The study was approved by the ethics committee of the hospital; all principles of the Declaration of Helsinki were followed. Written informed consent was obtained from all patients. Diabetes classification was based on the guidelines from the American Diabetes Association [25]. Demographic, clinical, laboratory results and medication data were collected from the clinical records.

### 2.2. Laboratory Measurements

Fasting blood samples were drawn from all subjects and plasma/serum was frozen at −80 °C until further analysis. Estimated glomerular filtration rate (eGFR), phosphate (P), calcium (Ca), magnesium (Mg), creatinine, parathyroid hormone (PTH), glycated hemoglobin (HbA1c), 1.25 dihydroxicolecalciferol (1.25(OH)2D3 vitamin D), tumour necrosis factor alpha (TNFα), intact fibroblast growth factor 23 (FGF-23), and soluble α-Klotho, were measured in serum/plasma as described [26,27,28]. The albumin to creatinine ratio in urine (ACR) was determined as described [26]. GFR was estimated according to the Chronic Kidney Disease Epidemiology Collaboration (CKD-EPI) Equation [29]. Serum levels of GRP were determined using the only available validated sandwich ELISA assay for the quantification of total GRP protein forms [22,24]. Briefly, total GRP ELISA is a sandwich dual antibody system using two GRP specific polyclonal antibodies, the capture antibody specifically recognizes the N-terminal of human GRP-F1 isoform, and the detecting antibody is directed against the C-terminal GRP sequence 54–74 (CTerm-GRP) (GenoGla Diagnostics) [22,24]. Inter- and intra-assay CV (%) were found as 4.46 and 5.03, respectively. Blinded measurements of GRP levels were performed at GenoGla Diagnostics, CCMAR, University of Algarve, Faro, Portugal. 

### 2.3. Blood Pressure

Systolic and diastolic blood pressure (BP) were determined with oscillometric methods, with the patient in dorsal decubitus. Three measurements were taken with an interval of 5 min.

### 2.4. Evaluation of Mitral and Aortic Valve Calcification

The assessment of valve calcification was performed using echocardiography, following the KDIGO guidelines. Accordingly, the Work Group suggests that in patients with CKD (G3a-G5D), an echocardiogram can be used to detect the presence or absence of VC as a reasonable alternative to CT-based imaging [30]. A good correlation between echocardiographic measurements of valve area and the valvular calcium Agatston score was previously demonstrated [31]. Echocardiographic evaluation was obtained using standard M mode and two-dimensional images (Vivid 7 Dimension-GE Healthcare Ultrasound; GE Healthcare, Waukesha, WI, USA). Offline analysis was obtained using the workstation Echopac PC’08 version 7.0.0 GE Vingmed Ultrasound (GE Healthcare), and the measurements were consistently obtained by the same physician. Images were digitally stored and analyzed by two independent experienced cardiologists. Quality control procedures included blind rereading and patient reexamination to allow assessment of intra-reader variability, inter-reader variability, and intra-patient variability. An average of three measurements was used for each variable. The subjects were categorized according to the presence of valvular calcification. The evaluation of the aortic valve calcification was performed using a semi-quantitative assessment as proposed by Lullo et al. [32]: non-calcified = score 1 (partial calcification on single cusp) and score 2 (partial calcification on two cusps); calcified = score 3 (extended calcification on two cusps) and score 4 (extended calcification on all three cusps). The extent of mitral valve calcification was determined according to the Wilkins calcification scores as: non-calcified = grade 1 and 2; calcified = grade 3 and 4 [33].

### 2.5. Statistical Analysis

Descriptive results were presented using mean and standard deviation (±SD) for continuous variables with normal distribution, using the Kolmogorov–Smirnov test. Two-sample *t*-tests were used to assess differences in subgroups defined by mitral valve calcified/non calcified and aortic valve calcified/non calcified for continuous measures. Subjects were split into two groups according to the median value of serum GRP: GRP ≥ 0.9 ng/mL and GRP < 0.9 ng/mL, to determine the percentage of calcified/non calcified mitral valve and calcified/non calcified aortic valve. Chi-square tests were used to test for association between mitral valve calcified/non calcified and aortic valve calcified/non calcified. Partial correlations were used to analyse relationships between aortic and mitral valve calcification with GRP and renal function (eGRF)**,** adjusted by sex and age groups. Univariate logistic regression analysis was used to identify independent factors associated with aortic and mitral valve calcification. Statistically significant variables were analysed in multivariate logistic regression models to assess the main predictive risk factors for aortic and mitral valve calcification. Potential confounding factors offered to the logistic regression models included age, gender, eGFR, FGF-23, TNFα, P, calcium x phosphate (CaxP), α-Klotho and GRP. The exponentials of the model parameters were the adjusted odds ratio (ORa) to other variables of the model, with 95% confidence interval. A modified Poisson regression with robust error variance estimation to calculate adjusted prevalence ratios (aPR) was used to estimate the cumulative relative risk of aortic and mitral valve calcification. Variables included in these multivariate analysis were age, eGRF, TNFα, calcium, phosphorus, CaxP, PTH, GRP, 1.25(OH)2D3 vitamin D, FGF-23 and α-Klotho. The receiver operating characteristic curves (ROC) were constructed to assess the sensitivity and specificity of risk factors for valvular calcifications. The median of risk factors was used to determine the best cut-off for the ROC curve. The null hypothesis was rejected below the level of 5%. Statistical analysis was performed with SPSS (version 17.0).

## 3. Results

This study enrolled 80 consenting diabetic patients meeting the inclusion criteria with stage 2–4 CKD (stage 2, n = 23; stage 3, n = 39; stage 4, n = 18), 28.7% females, mean age of 56 ± 8.1 years (range: 41–65). All variables had a normal distribution. The mean GRP levels was 0.9 ± 0.56 ng/mL (range, 0.19–2.6 ng/mL). The subjects were classified into groups according to the non-calcified or calcified valves (mitral valve calcified/non calcified and aortic valve calcified/non calcified). Table 1 describes the patients’ main clinical and biochemical characteristics, as a function of mitral and aortic valves calcification, including osteo-mineral markers and known risk factors for CVD. In total, 36.2% of patients presented mitral valves calcification (65.5% (n = 19) males and 34.4% (n = 10) females), and 36.2% had aortic valves calcification (72.4% (n = 21) males and 27.6% (n = 8) females). The groups with calcified mitral and aortic valves displayed significantly lower levels of eGFR (*p* < 0.0001 for both groups), GRP (*p* < 0.0001 for both groups), Mg (*p* = 0.029 and *p* = 0.001, respectively) and α-Klotho (*p* = 0.002 for both groups), while higher levels of P (*p* = 0.001 for both groups), PTH (*p* = 0.025 and *p* = 0.030, respectively), FGF-23 (*p* < 0.0001 for both groups) and TNFα (*p* = 0.037 for both groups), as compared to the respective non-calcified groups. No differences were found between groups regarding age, gender (f-m), hemoglobin, albumin, ACR, duration of disease, HgA1c, Ca, CaxP, 1.25(OH)2 Vitamin D and BP (Table 1).

**Figure 1:**
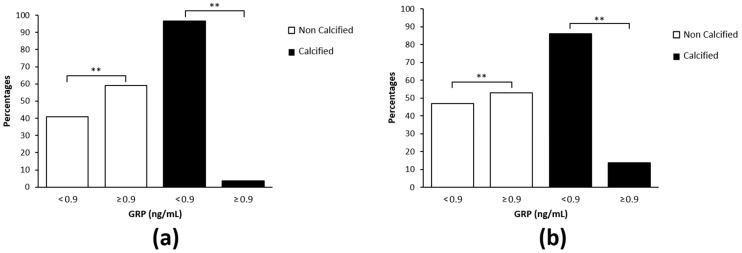
Association between Gla-rich protein (GRP) serum levels and valvular calcification. (**a**,**b**) Percentage of patients with calcified and non-calcified aortic (**a**) and mitral (**b**) valves, across the median GRP serum levels (<0.9; ≥0.9 ng/mL); the chi-square test was used to evaluate this association (** *p* < 0.0001).

The association between levels of GRP and aortic and mitral valves calcification using the chi-square test showed that higher levels of GRP were associated with a significant higher percentage of patients without aortic (96.7%) and mitral (87%) valves calcification, and to a lower percentage of patients with aortic (3.3%) and mitral (13%) valves calcification (Figure 1a,b, respectively).

**Figure 2:**
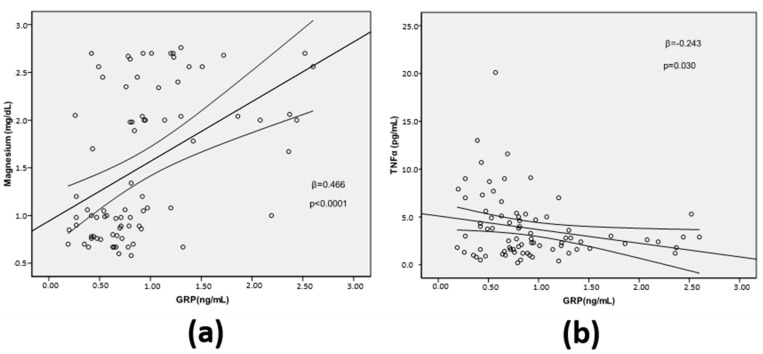
Association between Gla-rich protein (GRP) serum levels and magnesium (Mg) (**a**), and tumor necrosis factor alpha (TNFα) (**b**), using the simple linear regression analysis.

The relationships between GRP serum levels and Mg and TNFα, which were not previously reported for this cohort, were assessed using the simple linear regression. GRP serum levels were positively associated with levels of Mg (β = −0.466; *p* < 0.0001) and inversely associated with TNFα (β = −0.243; *p* = 0.03) (Figure 2a,b, respectively).

Partial correlations between GRP levels, mitral valves calcification and eGFR were analysed after adjustments for age and gender. A strong negative correlation was found between GRP and mitral valves calcification (r = −0.754, *p* < 0.0001), and a strong positive correlation with eGFR (r = 0.823, *p* < 0.0001), while a weak negative correlation was found between mitral valves calcification and eGFR (r = −0.421, *p* < 0.0001) (Table 2). Highly similar results were obtained for partial correlation analysis between GRP levels, aortic valves calcification and eGFR after adjustments for age and gender (Table 3).

Univariate logistic regression analysis was used to identify independent factors associated with aortic and mitral valves calcification (Table 4), and statistically significant variables were analysed in multivariate logistic regression models (Table 5). The results clearly show that lower levels of GRP and Mg are independent risk factors for mitral valve calcification (ORa = 0.268, *p* = 0.005; ORa = 0.747, *p* = 0.003, respectively) and aortic valve calcification (ORa = 0.202, *p* = 0.022; ORa = 0.580, *p* = 0.008, respectively) (Table 5). Additionally, high levels of P and FGF-23 were also found as independent risk factors for mitral valve calcification (ORa = 1.078, *p* = 0.001; ORa = 1.209, *p* = 0.035), and aortic valve calcification (ORa = 1.497, *p* = 0.002; ORa = 1.126, *p* = 0.011) (Table 5).

Furthermore, the Poisson regression analysis showed that low levels of GRP and Mg are cumulative risk factors for the occurrence of mitral valves calcification (aPR = 0.750; 95% CI 0.456–0.976; *p* = 0.024; aPR = 0.762; 95% CI 0.256–0.963; *p* = 0.028, respectively) and aortic valves calcification (aPR = 0.813; 95% CI 0.113–0.937; *p* < 0.0001; aPR = 0.809; 95% CI 0.391–0.974; *p* = 0.006, respectively) (Table 6). Additionally, high levels of P were associated with mitral (aPR = 1.110; 95% CI 1.001–2.803; *p* = 0.032) and aortic (aPR = 1.720; 95% CI 1.396–3.310; *p* = 0.002) valves calcification.

**Figure 3:**
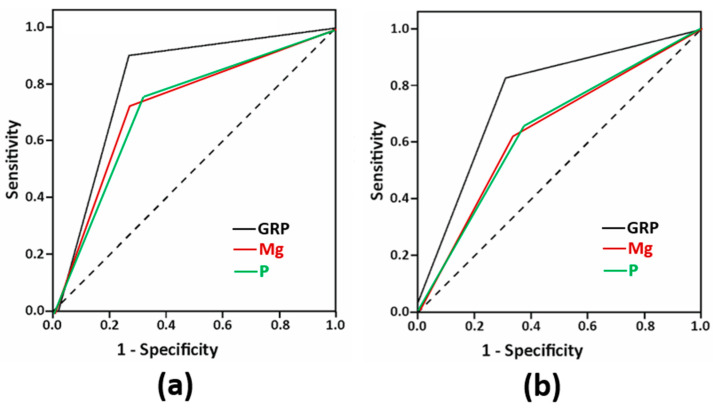
Receiver operating characteristic (ROC) curves of serum GRP, magnesium (Mg) and phosphate (P) for aortic valve calcification (**a**) and mitral valve calcification (**b**).

ROC curves were used to evaluate the diagnostic value of GRP, Mg and P for aortic and mitral valves calcification, and the results showed that the AUC of GRP for both aortic and mitral valves calcification were 0.802 ± 0.051, 95% CI (0.701–0.903), *p* < 0.0001 and 0.762 ± 0.056, 95% CI (0.653–0.872), *p* < 0.0001, respectively, which were higher than those of Mg and P in both aortic (0.725 ± 0.06 95% CI (0.607–0.843), *p* = 0.001 and 0.722 ± 0.060 95% CI (0.605–0.840), *p* = 0.001, respectively) (Figure 3a) and mitral (0.644 ± 0.065 95% CI (0.516–0.771), *p* = 0.033 and 0.641 ± 0.065 95% CI (0.515–0.768), *p* = 0.036, respectively) valves calcification (Figure 3b). Cutoff values were 0.9 ng/mL for GRP, 4 mg/dL for P, and 1.6 mg/dL for Mg.

## 4. Discussion

In this work we show that decreased levels of serum GRP are strongly associated with increased risk of aortic and mitral valve calcification in a population of adult diabetic patients with mild to moderate CKD. In addition, decreased levels of Mg and increased levels of P were also found independently associated with increased risk of both aortic and mitral valve calcification. Moreover, the simultaneous occurrence of decreased GRP and Mg and increased P levels are cumulative risk factors for valvular calcification (VC) in this population. Between these three factors, GRP showed the highest diagnostic value for aortic and mitral valves calcification. To our knowledge, this is the first clinical study showing an association between circulating GRP levels and valvular calcification. We recently reported that low levels of GRP were strongly associated with increased vascular calcification, pulse pressure and increased levels of the calcification promotors P, FGF-23 and CaxP, in this same patient cohort [24]. Here we show that GRP serum levels are positively associated with Mg, a known vascular calcification inhibitor [34,35]. The importance of P and Mg in vascular calcification and cardiovascular risk have been extensively demonstrated at both epidemiological and mechanistic levels, both in the general population and CKD, with hyperphosphatemia and hypomagnesia associated with increased vascular calcification, cardiovascular events and mortality [34,35,36,37,38,39]. In relation to VC, hyperphosphataemia has been associated with aortic and mitral valves calcification, including in moderate CKD patients [12,40,41,42,43]. Low serum Mg was found associated with the prevalence and incidence of aortic valve calcification in an adult population without known CVD and CKD [44]. In moderate CKD, low serum Mg was associated with abdominal aortic calcification, increased pulse pressure, mitral valve calcification and increased intima media thickness [45,46,47]. The findings that decreased levels of GRP and Mg and increased levels of P are independent and cumulative risk factors of VC, are in line with the current knowledge of their involvement on the pathophysiological mechanisms of cardiovascular calcification, and reinforce the crucial role of bone mineral metabolism in cardiovascular risk. Importantly, both GRP and Mg have been shown to inhibit high P-induced calcification of vascular smooth muscle cells (VSMCs), suggesting a tight and important relationship between these three factors. Mg status was shown to modify the risk of P-induced progression to end-stage kidney disease [48], also suggesting decreased levels of Mg and increased levels of P as cumulative risk factors in CKD progression.

Mechanistically, P is known to function as a primary stimulus for the osteochondrogenic differentiation of vascular and valvular cells [38,49]. Conversely, both GRP and Mg function as calcification inhibitors through several, and interestingly, coincident molecular processes, such as the inhibition of VSMCs osteochondrogenic differentiation through down-regulation of osteogenic and up-regulation of contractile markers, the inhibition of calciprotein particles (CPP) maturation, and capacity to act as anti-inflammatory agents [21,22,23,34,35,50,51].

Overall, these data clearly indicate GRP and Mg as essential protective factors in the calcification milieu, providing the biological rational for decreased levels of GRP and Mg as independent risk factors of VC. Additionally, although a relation between GRP and Mg at the cell level and possible interactions and/or intersections in their mechanisms of action are currently unknown, our findings that low levels of GRP and Mg and high levels of P are cumulative risk factors for VC, together with their simultaneous involvement in common cardiovascular calcification molecular processes, suggest a synergistic effect affecting cardiovascular calcification outcomes.

Our results showing a strong association between GRP and eGFR, as previously demonstrated [24], and with mitral and aortic VC, but a weaker association between eGFR and VC, suggest that GRP is involved in the pathophysiology of VC regardless of the stage of renal disease. This is in line with recent results showing an association between GRP and coronary artery calcification (CAC) in non-CKD patients with atrial fibrillation and heart failure [52].

Of note, therapies aiming to counteract hyperphosphatemia using phosphate binders have shown limited results in the suppression of CAC [53], while more recent clinical trials demonstrated that magnesium oxide is able to slow progression of CAC but not of thoracic aorta calcification [54]. This reinforces the notion that despite general assumption that mechanisms of calcification are similar within the vascular tree, specific factors can differently drive and affect calcification depending on the location, type of cells and environment, and that additional efforts are required to discover novel biomarkers and interventional agents to fight cardiovascular calcification. In calcified aortic valve disease, GRP was found highly accumulated at sites of mineral deposition and foam cells, and suggested to be associated with osteoblast-like VICs after myofibroblast-VIC differentiation [21], indicating a specific GRP action in VC.

It should be noted that in our study, although intact FGF23 was found as an independent risk factor of both aortic and mitral valves calcification, it was not identified as a cumulative risk factor for valves calcification. Many epidemiological studies have demonstrated that elevated levels of FGF23 are associated with renal function declining, increased cardiovascular morbidity and mortality, and higher aortic and coronary calcification scores [55,56,57,58]. However, a recent meta-analysis study suggests that there is no causal relation between FGF23 levels and cardiovascular risk, although this is still under investigation [59]. An important issue concerning the determination of FGF23 levels relates with the lack of consensus on a gold-standard method for dosing this hormone. FGF23 circulates as a full-length or intact protein (iFGF23), which constitutes the biologically active molecule, and as carboxi-terminal fragments (cFGF-23), whose activity remains controversial. ELISAs are available for both iFGF23 and cFGF23, but most of the published studies have only measured one of these forms. In some studies comparing iFGF23 and cFGF23, cFGF23 has been suggested as more sensitive in detecting eGFR decline in a non-CKD population [60], and increased risk of overall graft loss in kidney transplant recipients [61]. In addition, cFGF23 was suggested to mediate the association between iron deficiency and mortality in renal transplant recipients [62], and associated with red cell distribution width in CKD patients with heart failure [63]. In patients with atherosclerotic cardiovascular disease, levels of both iFGF23 and cFGF23 were associated with vascular calcification [64]. Clearly, additional comparative studies are required to further elucidate the different performances of iFGF23 and cFGF23 within specific populations and specific clinical features.

Major limitations of our study include the small sample size, requiring additional and larger studies to strengthen the evidence on the clinical relevance of GRP in VC, measurements of serum GRP at a single point, and the absence of reference intervals for GRP levels in a healthy population. Additionally, this study included subjects with mild to moderate CKD from a single centre, and may not be representative of kidney disease of other etiologies.

## 5. Conclusions

This study shows that decreased circulating levels of GRP and Mg and increased levels of P are independent and cumulative risk factors for mitral and aortic valves calcification in diabetic patients with moderate CKD. The high diagnostic value found for GRP in VC, together with previous knowledge that levels of GRP are also associated with vascular calcification and bone mineral metabolism [25], suggest GRP as a novel marker for cardiovascular calcification of potential clinical utility for cardiovascular risk assessment, calling for additional molecular and clinical research.

## 6. Patents

The tools and methods described in this manuscript are included in a Patent Cooperation Treaty (PCT) patent application PCT/PT2009000046.

## Figures and Tables

**Table 1:** Characteristics of the study population according to mitral and aortic valves calcification (n = 80).

General Characteristics	Mitral Valve		Aortic Valve	
	Calcified/ Non-Calcified	*p* Value	Calcified/ Non-Calcified	*p* Value
	n = 29/51		n = 29/51	
Age (years)	56.1 ± 9.8/60.2 ± 2.5	0.349	58.8 ± 8.7/56.6 ± 9.7	0.349
Gender (f–m)	10–19/14–37	0.340	8–21/16–35	0.463
BMI (Kg/m^2^)	24.16 ± 2.67/23.4 ± 4.20	0.450	24.07 ± 3.26/24.3 ± 2.03	0.289
Hb (g/dL)	12.93 ± 1.82/12.35 ± 1.52	0.234	12.87 ± 3.22/12.4 ± 2.5	0.230
Albumin (g/dL)	4.17 ± 1.72/4.14 ± 1.53	0.765	4.82 ± 1.52/4.14 ± 0.4	0.835
ACR (µg/mg)	155.2 ± 24.49/166.4 ± 2.3	0.650	153 ± 26.8/125.7 ± 17.4	0.745
eGFR (mL/min per 1.73 m^2^)	37.1 ± 14.7/53.0 ± 17.9	<0.0001	34.6 ± 11.19/54.4 ± 17.6	<0.0001
Phosphate (P) (mg/dL)	4.2 ± 0.6/3.8 ± 0.6	0.001	4.3 ± 0.6/3.7 ± 0.59	0.001
Calcium (Ca) (mg/dL)	9.3 ± 0.50/9.5 ± 0.6	0.119	9.3 ± 0.5/9.5 ± 0.6	0.119
Magnesium (Mg) (mg/dL)	1.2 ± 0.7/1.6 ± 0.7	0.029	1.16 ± 0.66/1.7 ± 0.74	0.001
PTH (pg/mL)	149.2 ± 10.4/104.5 ± 35.5	0.025	173.2 ± 78.9/91.2 ± 8.7	0.030
Calcium x Phosphate (CaxP) (mg^2^/dL^2^)	34.4 ± 5.66/35.6 ± 6.05	0.538	36.5 ± 6.2/35.6 ± 5.7	0.528
FGF-23 (RU/mL)	197.08 ± 20.9/90.3 ± 29.5	<0.0001	201.1 ± 10.1/97.8 ± 15.14	<0.0001
1.25(OH)2 Vitamin D (pg/mL)	20.7 ± 7.2/22.7 ± 7.6	0.312	22.5 ± 7.2/20.8 ± 7.2	0.311
GRP (ng/mL)	0.59 ± 0.28/1.1 ± 0.6	<0.0001	0.53 ± 0.22/1.1 ± 0.58	<0.0001
α-Klotho (pg/mL)	189.2 ± 71.7/319.6 ± 51.05	0.002	190.5 ± 80.2/318.4 ± 46.1	0.002
TNFα (pg/mL)	5.4 ± 1.9/3.1 ± 1.3	0.037	7.1 ± 1.8/3.07 ± 2.1	0.037
HbA1c (%)	7.25 ± 1.20/7.36 ± 1.61	0.518	6.06 ± 0.20/6.8 ± 0.3	0.329
Systolic BP (mmHg)	127 ± 9.8/127.2 ± 7.84	0.353	128.5 ± 8.8 /126.7 ± 8.8	0.353
Diastolic BP (mmHg)	74.7 ± 8.2/73.3 ± 8.14	0.323	73 ± 8.3/74.5 ± 3.4	0.371
Diabetes-related CKD evolution time (months)	12 ± 0.7/10 ± 0.8	0.123	12 ± 1.3/12.5 ± 2.5	0.992
RAS inhibitor/or ACEI (%)	78.7/21.3	0.068	50.7/47.3	0.184
Calcium channel blockers with renoprotective action (%)	35.6/64.4	0.052	48.6/32.4	0.151

BMI, body mass index; Hb, hemoglobin; ACR, urine albumin to creatinine ratio; eGFR, estimated glomerular filtration rate; PTH, parathyroid hormone; FGF-23, fibroblast growth factor 23; TNFα, tumor necrosis factor alpha; GRP, Gla-rich protein; HbA1c, glycated hemoglobin; BP, blood pressure; CKD, chronic kidney disease; ARA II, angiotensin II receptor antagonists; ACEI, angiotensinogen converting enzyme inhibitors.

**Table 2:** Partial correlation analysis between GRP, calcified mitral valve and estimated glomerular filtration rate (eGFR) after adjustments for age and gender.

Variables	GRP		Calcified Mitral Valve		eGFR	
	r	*p* Value	r	*p* value	r	*p* Value
GRP	1.00		−0.754	<0.0001	0.823	<0.0001
Calcified mitral valve	−0.754	<0.0001	1.00		−0.421	<0.0001
eGFR	0.823	<0.0001	−0.421	<0.0001	1.00	

Controlling variables: age and gender. Coefficient (r); two-tailed test of significance is used. GRP, Gla-rich protein; eGFR, estimated glomerular filtration rate.

**Table 3:** Partial correlation analysis between GRP, calcified aortic valve and estimated glomerular filtration rate (eGFR) after adjustments for age and gender.

Variables	GRP		Calcified Aortic Valve		eGFR	
	r	*p* Value	r	*p* Value	r	*p* Value
GRP	1.00		−0.786	<0.0001	0.823	<0.0001
Calcified aortic valve	−0.786	<0.0001	1.00		−0.525	<0.0001
eGFR	0.823	<0.0001	−0.525	<0.0001	1.00	

Controlling variables: age and gender. Coefficient (r); two-tailed test of significance is used. GRP, Gla-rich protein; eGFR, estimated glomerular filtration rate.

**Table 4:** Risk factors associated with mitral and aortic valves calcification.

Variables	Calcified Mitral Valve		Calcified Aortic Valve	
	ORa (95% CI)	*p* Value	ORa (95% CI)	*p* Value
Age	1.024 (0.974–1.077)	0.345	1.043 (0.977–1.256)	0.300
eGFR	0.945 (0.915–0.976)	0.001	0.900 (0.780–0.998)	<0.0001
TNFα	1.193 (1.019–1.397)	0.028	1.340 (1.056–1.500)	0.024
Ca	0.566 (0.267–1.197)	0.136	0.574 (0.272–1.213)	0.146
P	2.310 (1.111–4.803)	0.025	4.340 (1.004–8.745)	<0.0001
CaxP	1.022 (0.945–1.105)	0.585	1.026 (0.949–1.109)	0.522
PTH	1.003 (0.999–1.006)	0.155	1.006 (1.001–1.011)	0.024
Mg	0.489 (0.254–0.942	0.033	0.332 (0.161–0.682)	0.014
GRP	0.450 (0.234–0.657)	<0.0001	0.567 (0.367–0.905)	<0.0001
1.25(OH)2 Vitamin D	1.040 (0.974–1.110)	0.246	1.040 (0.974–1.110)	0.246
FGF-23	1.011 (1.005–1.017)	<0.0001	1.210(1.000–1.400)	<0.0001
α-Klotho	0.995 (0.992–0.998)	0.002	0.980 (0.880–0.990)	0.003

Univariate logistic regression analysis. ORa, adjusted odds ratio; CI, confidence interval; eGFR, estimated glomerular filtration rate; TNFα, tumour necrosis factor alpha; P, phosphate; Ca, calcium; CaxP, calcium x phosphate; PTH, parathyroid hormone; Mg, magnesium; GRP, Gla-rich protein; FGF-23, fibroblast growth factor 23.

**Table 5:** GRP is an independent risk factor associated with mitral and aortic valves calcification.

Variables	Calcified Mitral Valve		Calcified Aortic Valve	
	ORa (95% CI)	*p* Value	ORa (95% CI)	*p* Value
eGFR	0.995 (0.935–1.058)	0.865	0.902 (0.905–1.042)	0.440
TNFα	1.043 (0.843–1.245)	0.188	1.105 (0.929–1.401)	0.184
P	1.078 (1.000–1.612)	0.001	1.497 (1.004 -2.378)	0.002
GRP	0.268 (0.101–0.725)	0.005	0.202 (0.109–0.401)	0.022
Mg	0.747 (0.263–0.921)	0.003	0.580 (0.173–0.948)	0.008
FGF-23	1.209 (1.099–1.619)	0.035	1.126 (1.034–1.436)	0.011
α-Klotho PTH	1.002 (0.997–1.037	0.505	1.002 (0.995–1.009) 0.999 (0.890–1.002)	0.564 0.055

Multivariate logistic regression. ORa, adjusted odds ratio; CI, confidence interval; eGFR, estimated glomerular filtration rate; TNFα, tumour necrosis factor alpha; P, phosphate; Mg, magnesium; GRP, Gla-rich protein; FGF-23, fibroblast growth factor 23; PTH, parathyroid hormone.

**Table 6:** GRP, Mg and P are cumulative risk factors for valvular calcifications.

Variables	Calcified Mitral Valve				Calcified Aortic Valve			
	aPR	Robust Std. Err.	(95% CI)	*p* Value	aPR	Robust Std. Err.	(95% CI)	*p* Value
Age	1.023	0.0530	0.500–1.054	0.130	1.018	0.0175	0.987–1.050	0.263
eGFR	1.003	0.1310	0.978–1.015	0.813	0.999	0.0189	0.963–1.037	0.962
TNFα	1.035	0.0255	0.984–1.088	0.179	1.037	0.236	0.990–1.086	0.125
Ca	0.470	0.1297	0.304–1.105	0.136	0.752	0.3234	0.399–1.416	0.377
P	1.110	0.0352	1.001–2.803	0.032	1.720	0.3054	1.396–3.310	0.002
CaxP	1.011	0.0259	0.961–1.064	0.677	0.965	0.0262	0.917–1.016	0.179
Mg	0.762	0.2779	0.256–0.963	0.028	0.809	0.3712	0.391–0.974	0.006
PTH	1.000	0.0010	0.998–1.002	0.790	1.003	0.1008	0.901–1.124	0.071
GRP	0.750	0.0197	0.456–0.976	0.024	0.813	0.2430	0.113–0.937	<0.0001
1.25(OH)2 Vitamin D	1.020	0.0175	0.986–1.056	0.253	0.991	0.0126	0.967–1.016	0.489
FGF-23	1.002	0.0116	0.999–1.005	0.204	0.998	0.1021	0.994–1.003	0.258
α-Klotho	1.000	0.0015	0.996–1.002	0.818	1.000	0.0014	0.998–1.003	0.843

Robust Poisson regression model. aPR: adjusted prevalence ratios; 95% CI for aPR: 95% confidence interval for the prevalence ratios (PR); eGFR, estimated glomerular filtration rate; TNFα, tumour necrosis factor alpha; Ca, calcium; P, phosphate; CaxP, calcium x phosphate; Mg, magnesium; PTH, parathyroid hormone; GRP, Gla-rich protein; FGF-23, fibroblast growth factor 23.

## Data Availability

Data available on request due to privacy restrictions. The data presented in this study are not available, because they are in the process of analysis for results publication.
